# Exploring Predictors of Counselors’ Acceptance of Virtual Reality Exposure Therapy With Resistance and Job Contexts as Moderators: Cross-Sectional Mixed Methods Study

**DOI:** 10.2196/81803

**Published:** 2025-12-31

**Authors:** Myungsung Kim, Min Jeon, Yerin Lee, Sangil Lee, Hwang Kim, Dooyoung Jung

**Affiliations:** 1 Graduate School of Health Science and Technology, Ulsan National Institute of Science & Technology (UNIST), 201 Main Administration Bldg. B103, 50, UNIST-gil, Eonyang-eup, Ulju-gun, Ulsan, 44919, Republic of Korea, 82 52 217 2911; 2 School of Liberal Arts, Ulsan National Institute of Science & Technology (UNIST), Ulsan, Republic of Korea; 3 Department of Design, Ulsan National Institute of Science & Technology (UNIST), Ulsan, Republic of Korea; 4 Department of Biomedical Engineering, Ulsan National Institute of Science & Technology (UNIST), Ulsan, Republic of Korea

**Keywords:** virtual reality, exposure therapy, technology acceptance, digital mental health, counseling psychology, mixed methods, health technology adoption

## Abstract

**Background:**

Exposure therapy effectively treats anxiety disorders but faces implementation barriers, including cost, time constraints, and reluctance from therapists and clients. Virtual reality exposure therapy (VRET) offers a controlled digital alternative addressing these issues. However, adoption remains limited, with previous studies focusing mainly on hospital settings without considering individual or workplace factors.

**Objective:**

This study examined factors affecting counselors’ VRET acceptance across diverse settings. We used the Unified Theory of Acceptance and Use of Technology (UTAUT) extended with job stress and resistance to change. Open-ended questions provided a deeper understanding of counselors’ perspectives on VRET.

**Methods:**

A cross-sectional mixed methods study was conducted with 258 certified counselors across various settings, including universities, public institutions, and private clinics. Participants watched a 4-minute VRET introduction video and completed a survey measuring UTAUT variables (performance expectancy, effort expectancy, facilitating conditions, and social influence), resistance to change, and job stress. Stepwise forward selection multiple linear regression with moderation analyses was conducted to identify key predictors and test interaction effects. Open-ended responses (N=257, 290 meaning units) on VRET applicability and improvement suggestions were analyzed using team-based thematic analysis with iterative consensus coding.

**Results:**

Performance expectancy (*β*=.404, 95% CI 0.297-0.512, *P*<.001) and social influence (*β*=.387, 95% CI 0.280-0.494, *P*<.001) significantly predicted VRET adoption intentions (*R*^2^=0.494). Moderation analysis revealed that routine seeking weakened performance expectancy impact (*β*=–.160, 95% CI –0.277 to –0.043, *P*<.01), low job control strengthened it (*β*=.162, 95% CI 0.280-0.494, *P*<.005), and high job demands reduced social influence effects (*β*=–.150, 95% CI –0.263 to –0.036, *P*=.01). The narrow confidence intervals indicate precise estimation of these moderation effects. Younger counselors were more sensitive to contextual moderators, while older counselors prioritized performance expectancy. Thematic analysis identified 3 themes: counselor evaluation criteria for VRET, emphasizing content diversity and scientific validation; considerations for promoting and introducing VRET to counselors, addressing implementation challenges; and areas requiring continuous improvement for VRET field implementation, emphasizing professional competence and system reliability.

**Conclusions:**

This study advances VRET acceptance research by examining certified counselors across diverse nonhospital settings—unlike prior hospital-focused physician studies—and extending UTAUT with profession-specific moderators. Performance expectancy and social influence emerged as primary predictors, with routine seeking and job context significantly moderating these effects across age groups. Thematic analysis revealed that counselors evaluate VRET as a supplementary tool requiring scientific validation, diverse content, and structured training rather than technological usability alone. Findings inform practical strategies as follows: disseminating effectiveness evidence, leveraging professional networks, addressing work environment barriers for high-demand contexts, and developing age-appropriate approaches. Insights guide content developers, policymakers, and researchers implementing VRET beyond hospital settings.

## Introduction

### Background

Considered a leading standard treatment for anxiety and related disorders [1], exposure therapy is nonetheless seldom provided [2,3]. Time and financial constraints are often cited as principal reasons that hinder this well-known and efficient therapy from being prioritized by practitioners [4]. In addition, patients fear and avoid the situations they are asked to experience, inhibiting counselors’ willingness to recommend the therapy, further complicating its practical delivery [5]. To address and improve the negative perception of exposure therapy among practitioners, extensive research has shown that even simple educational interventions can enhance its implementation and use [6].

Moreover, current technological progress impacts exposure therapy and is reshaping its traditional application through the emergence of new resources. For instance, virtual reality (VR) technology, accessed through head-mounted displays, provides an immersive experience similar to real-life situations and has been used in controlled environments [7]. These rapid advancements in virtual reality exposure therapy (VRET) enable controlled and immersive exposure without the risks associated with real-world environments. A study involving university students has found that a VR plaza desensitization exercise significantly reduced the symptoms of agoraphobia and subjective discomfort compared with a control group [8]. Another study in a school context has shown that anxiety-inducing exposure significantly improves social anxiety symptoms [9]. Additionally, research has highlighted that in a virtual environment job interview scenario, participants’ anxiety increases when avatars are more realistic, mirroring effects similar to real-world exposure [10]. Botella et al [11] have also supported that VRET could reduce the symptoms of other phobic behaviors not directly targeted by exposure therapy, suggesting additional therapeutic potential.

Notably, VRET bears certain economic advantages, demonstrating cost-effectiveness compared with traditional exposure therapy [12]. Furthermore, systematic reviews have suggested that VRET could lead to future cost savings with the advancement of VR technology [13]. In recent years, VR costs and usability barriers have significantly decreased with the emergence of standalone wireless devices, such as the Oculus Quest. These affordable headsets provide high-quality immersive experiences without requiring complex setups, thereby rendering VRET more feasible in routine clinical contexts [14].

However, VRET may not always be perceived as cost-effective, but rather as a new intervention requiring additional investment by health practitioners [15]. The high setup complexity and initial costs necessary for VR implementation have been widely criticized. Therapists also experience challenges when adopting new technologies [16], and their perceptions strongly influence clinical adoption [17,18]. Importantly, simultaneous VR education for patients and providers has been shown to improve participation rates [19], and prior VR use [20], particularly VRET experience, positively influences therapists’ perceptions. A study in intensive care units, for instance, revealed that both providers and patients lacked the readiness and technical competence necessary for VR use, highlighting practical barriers to implementation.

Yet, despite technical advancements and growing affordability, the use of technology in clinical settings remains limited. Reported concerns regarding limited patient applicability, perceived inefficiency due to setup time or technical challenges, and side effects such as motion sickness continue to hinder adoption [21]. Although issues related to cost and motion sickness may diminish with continued technological progress [22,23], understanding how therapists perceive VRET and identifying ways to improve it remain crucial for its broader implementation. Currently, studies provide only limited insight into the factors that discourage or facilitate the adoption of these technologies. Therefore, understanding the factors affecting therapists’ acceptance of advanced technologies has become an important area of research.

The factors influencing VRET adoption align with the Unified Theory of Acceptance and Use of Technology (UTAUT), which includes performance expectancy, effort expectancy, facilitating conditions, and social influence. In the VRET context, these constructs are shaped by the therapist’s knowledge and experience with VR and by their perceived readiness to deliver such therapies. For example, loyalty to established practices and a lack of resources to implement changes have been identified as major challenges in training programs for evidence-based therapies [24]. Organizational factors also play a key role in determining a therapist’s approach or willingness to use technology [25]. Despite this, existing studies have largely focused on therapists’ perceptions without considering individual traits such as resistance to change and work environment characteristics [26].

Furthermore, previous studies predominantly targeted medical doctors or staff in hospital environments. According to the National Center for Mental Health in South Korea, 459 out of 545 psychiatric specialists are concentrated in general hospitals [27], and VRET is typically provided in only a few large hospitals. This study, therefore, seeks to expand the scope of VRET beyond traditional clinical environments by targeting members of the Korean Counseling Psychology Association who work in diverse contexts, including universities, public institutions, corporate counseling centers, and private practices.

This study addresses critical gaps in VRET acceptance literature by examining counselors across diverse nonhospital settings, extending UTAUT with profession-specific moderators (resistance to change, job stress), and conducting age-stratified analysis to capture hierarchical organizational dynamics in Korean counseling practice. Finally, acknowledging that therapists’ prior VR knowledge strongly affects acceptance and data validity [28], this study restricted VRET to panic disorder and social anxiety scenarios and included an introductory video to ensure informed feedback. The video included definitions, purposes, client perspectives, clinical evidence, and demonstrations of VRET use. In addition, respondents rated the importance and satisfaction of the video components to provide a comprehensive assessment of the VR introduction content.

### Study Objectives

This study examines counselors’ acceptance of VRET in Korea using the UTAUT. Given the absence of real-world VRET implementation in Korean counseling practice, the focus is on identifying the most influential predictors of acceptance intention rather than testing all theoretical relationships simultaneously. We examine associations between UTAUT constructs (performance expectancy, effort expectancy, facilitating conditions, and social influence) and acceptance, while also considering the moderating effects of prior VR experience, resistance to change, and work environment factors. To achieve this, the study (1) identified key predictors through stepwise regression analysis, (2) examined the moderating role of individual traits and work environment, (3) explored age-related differences in the hierarchical structure of Korean counseling organizations, and (4) conducted thematic analysis to provide contextual understanding through quotations and examples. The findings are intended to inform practical strategies for implementing VRET beyond hospital settings in Korean counseling practice.

## Methods

### Theoretical Model

With the advent and rapid advancement of digital technology, in which VR represents a significant component, the adaptation of traditional Technology Acceptance Models (TAMs) has become a central concern among researchers [29]. In response to this need, the UTAUT has evolved to accommodate these developments and has been widely used to investigate various forms of digital technology adoption, establishing itself as a robust and versatile theoretical framework [30].

Building on this framework and as explained above, the UTAUT model best fits the aims of this study. Compared with more traditional TAMs, UTAUT allows for the inclusion or extension of factors according to the characteristics of the population or technology under investigation. Supporting this application, several studies have expanded the scope of the 4 main factors previously mentioned (performance expectancy, effort expectancy, social influence, and facilitating conditions). In this regard, Staeck et al [31] identified “therapeutic alliance,” along with performance expectancy and social influence, as a major determinant influencing technology acceptance of e-mental health among psychotherapists. Other UTAUT-based studies have incorporated additional factors such as perceived risk, self-efficacy, and attention [32,33].

In our research, and informed by previous studies suggesting that therapists tend to be relatively conservative in adopting new technologies [28,34], resistance to change was included here as a moderator. Furthermore, based on evidence indicating that workload and autonomy influence innovation adoption [35], low job control and high job demands were also introduced as moderators. These inclusions aim to capture contextual and individual variables that may affect counselors’ acceptance of VRET.

In the original UTAUT framework, facilitating conditions are typically predictors of actual use behavior rather than behavioral intention. However, since VRET is not yet available in Korean counseling settings, we examined the relationship between facilitating conditions and behavioral intention. This methodological choice aligns with other pre-implementation technology acceptance studies and provides an appropriate basis for accurately interpreting our results without extrapolating to decision-making processes that are not applicable in this context [30,31].

**Figure 1.**
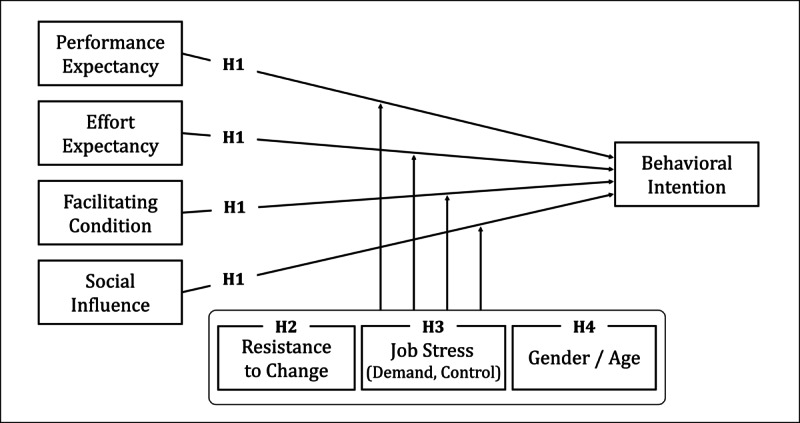
Theoretical framework for cross-sectional mixed methods study examining virtual reality exposure therapy (VRET) adoption among certified Korean counselors (n=258) working in universities, public institutions, and private clinics across South Korea (2023). The study investigates predictors of VRET acceptance for treating panic disorder and social anxiety using the Unified Theory of Acceptance and Use of Technology (UTAUT) extended with profession-specific moderators. The model illustrates hypothesized relationships between UTAUT constructs (performance expectancy, effort expectancy, facilitating conditions, and social influence), moderating factors (resistance to change: routine seeking, emotional reaction, short-term thinking, and cognitive rigidity; job stress: high job demands and low job control; demographics: age and gender), and behavioral intention to adopt VRET in counseling practice.

Integrating these theoretical considerations and extended variables, this study proposes the following hypotheses: (H1) Performance expectancy, effort expectancy, facilitating conditions, and social influence will positively predict counselors’ behavioral intention to adopt VRET; (H2) Resistance to change factors (routine seeking, emotional reaction, short-term thinking, and cognitive rigidity) will moderate the relationships between UTAUT predictors and behavioral intention; (H3) Job stress factors (high job demands and low job control) will moderate the relationships between UTAUT predictors and behavioral intention; and (H4) Demographic factors (gender and age) will moderate the relationships between UTAUT predictors and behavioral intention. The theoretical model is illustrated in Figure 1.

### Participants

The web-based professional communities hosted by the Korean Clinical Psychology Association and the Korean Counseling Psychology Association were selected as the primary recruitment channels. Email invitations were distributed through these platforms to reach potential participants. A convenience sampling approach was used, as participants self-selected to respond to recruitment announcements distributed through the professional associations’ web-based communities. Following recruitment, Google Forms was used to distribute the survey. Ethical and methodological considerations for reliable survey design were carefully followed, including an overview of the study’s purpose, confidentiality assurances, and informed consent. Finally, participants were provided with a link to a 4-minute introductory video on VRET.

For their participation, we provided Starbucks’ gift cards, each valued at approximately US $7 (KRW 10,000), as an incentive.

### Settings and Design

This study used an embedded mixed methods, cross-sectional design, with quantitative methods prioritized to address the primary research questions, while qualitative data provided complementary and expansionary insights. In line with the embedded mixed methods design, both quantitative and qualitative data were collected remotely and simultaneously within a single survey instrument that integrated closed and open-ended items. To ensure the avoidance of bias associated with this approach, the survey was pretested prior to distribution. Additionally, a panel of researchers reviewed the instrument and provided feedback on its structure, clarity, logical articulation, and content relevance.

This study adheres to established reporting standards for methodological transparency and quality. The quantitative and qualitative methods are reported in accordance with the Good Reporting of A Mixed Methods Study (GRAMMS) guidelines [36] (Checklist 1), and the qualitative strand specifically follows the Consolidated Criteria for Reporting Qualitative Research (COREQ) checklist [37] (Checklist 2). These frameworks ensure comprehensive reporting of study design, data collection procedures, analysis methods, and interpretation processes.

Along with basic demographic information, the survey was designed according to the objectives of the study, gathering details about participants’ counseling experience, types of counseling certifications held, and the primary age range of their clients. After watching the 4-minute introductory video on VRET, participants completed items based on the UTAUT model, which assessed their behavioral intention to use VRET, as well as VRET-related performance expectancy, effort expectancy, facilitating conditions, and social influence. Additional items evaluated work-related stress and resistance to change. Finally, open-ended questions explored participants’ views on the applicability and potential improvements of VRET. The survey required approximately 20 minutes.

To determine the appropriate sample size for multiple regression analysis, we used G*Power software to calculate the parameters. As the adoption of VRET among counselors is relatively unexplored, we assumed a medium effect size (Cohen f^2^=0.15), an alpha level of 0.05, and a desired power of 0.80. The number of predictor variables was set to 12, including the 4 main UTAUT predictors: age, sex, 2 job stress factors, and 4 resistance to change factors. The calculated minimum sample size was 127; however, to allow for subgroup comparisons by sex or age and accounting for the number of variables present, we aimed to collect data from a double sample of 254 participants.

### Introduction Video

**Figure 2.**
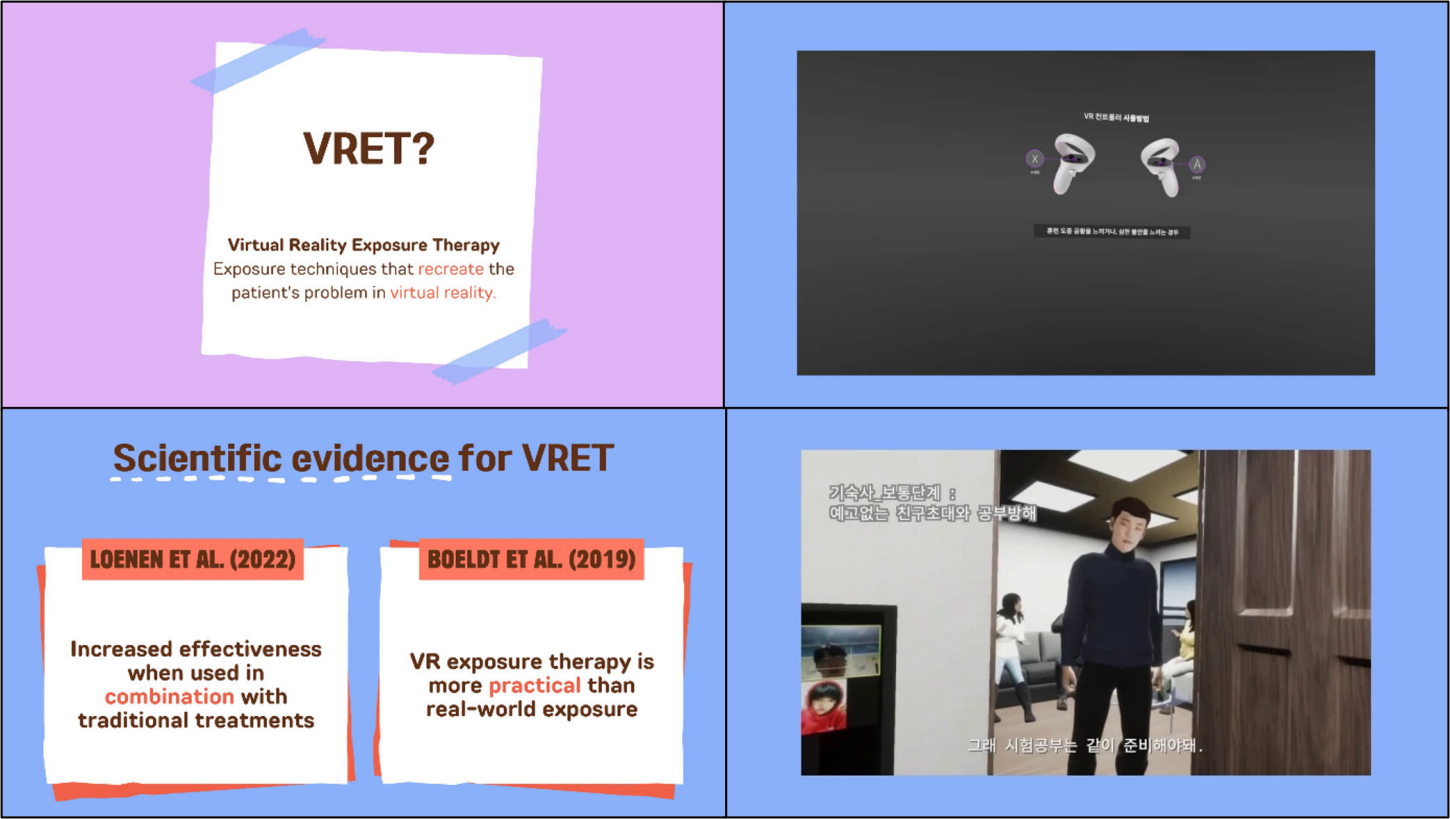
Four-minute Korean-language educational video shown to all participants (n=258) before survey completion to ensure baseline understanding of VRET. Content structure: (top left) definition of VRET, (top right) client-perspective VR interface, (bottom left) scientific evidence and clinical outcomes, (bottom right) actual exposure content for panic disorder and social anxiety scenarios. The complete video also includes explanations of therapeutic mechanisms and practical demonstrations of clinical implementation. Image translated to English for publication. The original Korean version is provided in Multimedia Appendix 1. VRET: virtual reality exposure therapy.

To ensure that participants unfamiliar with VRET achieved a minimum level of understanding, the video was provided before the survey completion and covered 5 main aspects in the following order: the definition of VRET, its purpose, sample content viewed by clients, scientific evidence (including clinical outcomes), and usage demonstrations. Two scenarios were included, one illustrating panic disorder exposure in open spaces and another depicting social anxiety exposure through social interactions. This material was specifically designed by the research team for a separate study and was produced in Korean. The complete video content is illustrated in Figure 2.

### Measures

Items related to the UTAUT model were modified to suit the context of this study, specifically targeting VRET, and are listed in Textbox 1. Behavioral intention was measured using 3 items that assessed the intention to incorporate VRET into practice. Performance expectancy was measured as the extent to which the participants anticipated that VRET would enhance their therapeutic outcomes. Effort expectancy included items measuring the anticipated effort to integrate VRET, considering the therapist’s and client’s efforts. Facilitating conditions addressed required VRET resources, such as cost, technical support, and infrastructure. Social influence examined the perceived acceptance and influence of VRET among clients, colleagues, and professional associations.

Resistance to change and work-related stress were moderators. The resistance to change scale assessed individual tendencies to resist new practices and was structured into 3 factors: routine seeking, emotional reactions, and short-term thinking [38]. Routine seeking reflects a preference for stability and a low level of novelty. Emotional reactions capture stress and tension that occur when unexpected changes happen. Short-term thinking reflects a tendency to focus on immediate inconveniences rather than potential long-term benefits. These tendencies were measured using 17 items rated on a 6-point Likert scale (1=strongly disagree, 6=strongly agree), translated into Korean to suit the context of this study. Work-related stress items were obtained from subscales of the Korean Occupational Stress Scale, which specifically measured job demands and low job control [39]. Job demands refer to the degree of work-related burden, encompassing time constraints, workload, and responsibility. Low job control reflected the level of autonomy in decision-making and discretion in job-related tasks.

Textbox 1. **Survey items measuring virtual reality exposure therapy (VRET) adoption predictors were adapted from the Unified Theory of Acceptance and Use of Technology framework. Constructs include behavioral intention (3 items), performance expectancy (5 items), effort expectancy (5 items, 2 excluded), facilitating conditions (2 items, 1 excluded), and social influence (3 items), all rated on 5-point Likert scales (1=strongly disagree, 5=strongly agree). Additional open-ended questions explored counselors’ perspectives on VRET applicability and improvement areas. Please note that items were excluded after reliability analysis (Cronbach α, factor loadings <.60).**

**Behavioral intention**

- If virtual reality (VR) devices and exposure therapy content are provided, I would be willing to use them in psychological therapy.
- If possible, I would incorporate VR exposure therapy content into my therapeutic process.
- In the future, I intend to apply VR exposure therapy content to clients.

**Performance expectancy**

- VR exposure therapy content will be useful in the psychological therapy process.
- VR exposure therapy content will alleviate clients’ anxiety-related symptoms.
- VR exposure therapy content will aid my work as a therapist.
- VR exposure therapy content will increase client satisfaction with psychological therapy.
- VR exposure therapy content will enhance clients’ engagement in therapy.

**Effort expectancy**

- Most clients will have the necessary skills to use VR exposure therapy content.
- It will not be difficult for clients to learn how to use VR exposure therapy content.
- The information provided by VR exposure therapy content will be easy for clients to understand.
- Most therapy providers will have the necessary skills to offer VR exposure therapy content.
- It will not be difficult for therapy providers to learn how to use VR exposure therapy content.
- Charging and managing VR devices to provide VR exposure therapy content will not be difficult (excluded).
- Providing various VR exposure therapy contents according to clients’ symptoms and levels will not be difficult (excluded).

**Facilitating conditions**

- I have the resources (budget, space) necessary to use VR exposure therapy content.
- If I encounter difficulties in using VR exposure therapy content, I will be able to seek help from others.
- VR exposure therapy content can be used alongside other therapeutic techniques I employ (excluded).

**Social influence**

- Clients will not have a negative attitude toward the use of VR exposure therapy content.
- Psychological therapy-related organizations and associations will recommend the use of VR exposure therapy content.
- My peers in therapy provision will recommend the use of VR exposure therapy content.

**Feedback on VRET (open-ended questions)**

- Please describe the overall applicability or areas for improvement for VR exposure therapy content.

### Ethical Considerations

This study was approved by the Institutional Review Board of the Ulsan National Institute of Science and Technology (approval number UNISTIRB–23‐057–A). This approval underscores the study’s commitment to uphold ethical standards that protect participants’ rights and safety throughout the research process.

Before participating, all participants received a detailed web-based description of the study, including its purpose, procedure, and potential risks. Participants provided informed consent through Google Forms, confirming their understanding of the study and willingness to participate. Considering that this study focused on analyzing perspectives on therapy rather than direct intervention, the participants’ mental health status was not assessed.

Survey data were anonymized to ensure confidentiality and privacy. Responses were collected via Google Forms, which was configured not to record digital identifiers such as IP addresses. Email addresses were collected separately solely for gift card distribution purposes, were not linked to survey responses during data storage or analysis, and were permanently deleted immediately after compensation distribution as disclosed in the informed consent. During data processing, each participant was assigned a unique numerical identifier (P1-P258) to ensure complete anonymity. The participants were informed that their data would be used solely for research purposes. The consent process emphasized their right to withdraw from the study at any time without penalties. Participants received a Starbucks gift card valued at approximately US $7 (KRW 10,000) as compensation for their time and participation. No images or materials in this manuscript contain identifiable individual participants, as only aggregate statistical data and anonymous quotations were used in reporting results.

### Analysis

#### Quantitative Model Analysis

Stepwise multiple linear regression with forward selection was used as the primary statistical method due to its exploratory capability in identifying the most influential predictors in this previously unstudied context. This approach was chosen over full model testing because our primary objective was to identify which UTAUT factors most strongly predict VRET acceptance intention in Korean counseling practice, providing practical guidance for implementation rather than confirming all theoretical relationships simultaneously. This approach is consistent with other exploratory technology acceptance studies that have used stepwise regression to identify key predictors of behavioral intention in health care and technology adoption contexts [40-42]. We first assessed the significance of the correlations between the independent and dependent variables by adding the most significant variables (*P*<.05) to the model. Based on the selection of the main effects, the moderating effects of gender, age, job stress, and resistance to change were analyzed.

Participants were divided into 3 groups of similar sizes by age to explore how the major predictors and moderators vary across life stages for 2 reasons. First, in Korean counseling organizations, age strongly correlates with professional roles and decision-making authority. Younger counselors typically work under supervision, whereas older counselors hold senior positions with greater autonomy. Second, existing technology acceptance research has shown that predictors of adoption and moderating effects vary significantly across age groups [43]. Age was treated as a continuous variable, and participants were classified into 3 groups for comparative analysis. All regression analyses were performed using Google Colab and the Scikit-learn (Sklearn) library.

#### Thematic Analysis for Open-Ended Responses

For open-ended responses, thematic analysis was performed using the qualitative data analysis tool QDA Miner. The open-ended questions explored participants’ general perceptions of VRET content and potential improvements for content targeting panic and social anxiety. Unlike traditional interview-based studies that pursue sequential saturation, our predetermined large sample (n=258) provided comprehensive perspective coverage, significantly exceeding the typical qualitative sample sizes of 6‐12 interviews [44]. Sample adequacy was established through systematic consensus-building among multiple coders and frequency-based validation criteria.

Following team-based consensus approaches [45], 3 researchers (MK, MJ, and YL) systematically analyzed responses through five steps: (1) segmenting 258 responses into 487 individual meaning units since participants often addressed multiple themes per response, (2) developing preliminary thematic categories from grouped similar concepts, (3) iteratively coding meaning units with continuous codebook updates through regular consensus meetings, (4) finalizing themes when no further updates were needed, with clear definitions and representative quotations for each code, and (5) applying a 4% frequency threshold (≥11 participants) for theme retention to enhance generalizability while maintaining professional relevance through team discussion.

The analysis was led by the first author (MK), a fourth-year graduate student in digital health care with previous qualitative research experience in social chatbot and behavioral intervention studies. The second author (MJ) and third author (YL), second and first-year digital health care graduate students, respectively, received comprehensive training in qualitative methodology through recommended textbooks and exemplar papers provided by the lead coder. All coding steps involved consensus among all 3 coders, eliminating the need for quantitative intercoder reliability calculations [45].

The thematic analysis aimed to identify factors not fully captured in the quantitative UTAUT analysis, particularly contextual interpretations of effort expectancy and facilitating conditions, and to provide a deeper understanding of the quantitative findings. Codes directly reflecting quantitative results (eg, performance expectancy predicting adoption intention) were minimally incorporated into higher-level themes to avoid redundancy and focus on novel insights complementing the quantitative analysis.

#### Missing Data

The survey was designed with mandatory response fields for all quantitative items to minimize missing data. All 258 participants who initiated the survey completed all required quantitative measures, resulting in no missing data for the primary analyses.

### Demographic Information

In total, 258 counseling professionals participated in the survey, and no participants were excluded from the analysis. Among them, 86.8% (224/258) were female, closely matching the 81.3% female representation among counselors and youth guidance professionals reported by the South Korean Ministry of Employment and Labor. The average age of participants was 42.17 years (SD 8.84), and more than half (163/258, 63.2%) had 10 or fewer years of counseling experience. The primary client age group reported by counselors was young adults (aged 19‐29 y), accounting for 39.2% (101/258), followed by middle-aged adults and adolescents. Most participants held a Level 1 or Level 2 counseling psychology certification from the Korean Counseling Psychological Association. The demographic characteristics of the participants are presented in Table 1.

**Table 1.** Demographic characteristics of certified Korean counselors participating in cross-sectional mixed methods survey examining virtual reality exposure therapy acceptance (n=258, South Korea, 2023). Participants were recruited via convenience sampling from the Korean Clinical Psychology Association and Korean Counseling Psychological Association web-based communities. Sample: mean age 42.17 years (SD 8.84, range 25‐64), 86.8% (224/258) female, 63.2% (163/258) with ≤10 years counseling experience. Counselors worked across diverse settings, including universities (educational institutions), public institutions (government-funded counseling centers), and private clinics. Most held Level 1 or 2 counseling psychologist certification from the Korean Counseling Psychological Association; multiple certifications common as Korean psychological counseling credentials include both national (eg, Korean Psychological Association, Ministry of Health and Welfare) and private professional certifications.

Variables	Participants
**Sex, n (%)**	
Female	224 (86.8)
Male	34 (13.2)
**Age (years)**	
Mean (SD)	42.17 (8.84)
Median (range)	42 (25-64)
**Years of counseling experience, n (%)**	
1 to 5	77 (29.8)
6 to 10	86 (33.3)
11 to 15	52 (20.2)
16 to 20	27 (10.5)
Over 20	16 (6.2)
**Primary client age range (years), n (%)**	
All age groups	27 (10.5)
Children (6 to 12)	8 (3.1)
Adolescents (13 to 18)	49 (19.0)
Young adults (19 to 29)	101 (39.2)
Middle-aged adults (30 to 49)	68 (26.4)
Older adults (50 to 64)	5 (1.9)
Seniors (65 and older)	0 (0)
**Counseling certification or license** ^**a**^ **, n (%)**	
Counseling psychologist certificate, Level 1^b^	75 (29.1)
Counseling psychologist certificate, Level 2^b^	175 (67.8)
Professional counselor certification, Level 1^c^	8 (3.1)
Professional counselor certification, Level 2^c^	14 (5.4)
Youth counselor, Level 1^d^	28 (10.9)
Youth counselor, Level 2^d^	136 (52.7)
Youth counselor, Level 2^d^	38 (14.7)
National certified clinical psychologist, Level 1^e^	10 (3.9)
National certified clinical psychologist, Level 2^e^	27 (10.5)
Clinical psychologist specialist^f^	5 (1.9)

a In South Korea, psychological counseling certifications are categorized as national and private certifications. The survey targeted individuals with at least one nationally accredited certification, such as those from the Korean Psychological Association and Korean Counseling Association. Counselors had more than one certification, with many having 2 or more.

b Korean Counseling Psychological Association. Levels 1 and 2 for professional and counseling psychologists, respectively.

c Korean Counseling Association.

d Korean Ministry of Gender Equality and Family.

e Korean Ministry of Health and Welfare.

f Korean Clinical Psychology Association.

## Results

### Descriptive Statistics

The reliability of the quantitative measures was assessed using Cronbach α. Most of the measures demonstrated acceptable reliability levels compared with previous studies; however, specific items were refined based on factor-loading values. These items included items 6 and 7 for effort expectancy; item 3 for facilitating conditions; routine seeking items 1 and 4; emotional reaction item 4; short-term thinking items 1 and 3; cognitive rigidity items 1 and 3 for resistance to change; and items 1, 2, and 3 for low job control. The refined reliability scores and item counts for each measure, with their sources, are listed in Table 2.

**Table 2.** Means, SDs, item counts, and internal consistency (Cronbach α) for all study measures: behavioral intention (dependent variable), Unified Theory of Acceptance and Use of Technology (UTAUT) predictors (performance expectancy, effort expectancy, facilitating conditions, and social influence), resistance to change moderators (routine seeking, emotional reaction, short-term thinking, and cognitive rigidity), and job stress moderators (high job demand and low job control). All scales were rated on multi-point Likert scales; retained scales demonstrated acceptable reliability (*α*≥.65).

Construct	Measurement	Mean (SD)	Items, n	Cronbach α
**Acceptance variable**				
Behavioral intention	UTAUT^a^	4.01 (0.76)	3	.91
**Predictors**				
Performance expectancy	UTAUT^a^	4.00 (0.62)	5	.89
Effort expectancy	UTAUT^a^	3.51 (0.68)	5	.86
Facilitating conditions	UTAUT^a^	2.81 (0.92)	2	.65
Social Influence	UTAUT^a^	3.39 (0.72)	3	.77
**Moderators**				
Routine seeking	Resistance to change^b^	3.36 (1.24)	3	.78
Emotional reaction	Resistance to change^b^	4.12 (1.10)	3	.81
Short-term thinking	Resistance to change^b^	3.08 (1.18)	2	.66
Cognitive rigidity	Resistance to change^b^	3.31 (0.62)	2	.65
High job demand	Job stress^c^	2.57 (0.59)	8	.84
Low job control	Job stress^c^	1.92 (0.42)	2	.68

a UTAUT (Unified Theory of Acceptance and Use of Technology) [30]: Four major factors of the UTAUT model were adapted to suit the context of virtual reality exposure therapy, whereas survey items were developed accordingly. Based on reliability analysis (Cronbach α and loading values), items 6 and 7 for effort expectancy and item 3 for facilitating conditions were refined.

b Resistance to change [38]: Based on the reliability analysis (Cronbach α, loading values), routine seeking items 1 and 4, emotional reaction item 4, short-term thinking items 1 and 3, and cognitive rigidity items 1 and 3 were refined.

c Job Stress: The Korean occupational stress scale [39] was used to measure stress related to job demands and lack of job autonomy, with items 1, 2, and 3 of the lack of autonomy subscale refined based on reliability analysis.

The average score for Intent to Use, which reflected the participants’ acceptance of VRET, was relatively high (4.01/5). Among the major UTAUT predictors, performance expectancy (4.00/5), effort expectancy (3.51/5), and social influence (3.39/5) were above the midpoint, while facilitating conditions were slightly below the midpoint (2.81/5).

Regarding the moderators, emotional reaction among the resistance to change factors was above the midpoint (4.12/6), whereas routine seeking, short-term thinking, and cognitive rigidity scored slightly below the midpoint (3.36/6, 3.08/6, and 3.31/6, respectively). High job demands and low job control averaged at the midpoint and slightly below the midpoint, respectively (2.57/4 and 1.92/4), indicating moderate job demands and relatively good job autonomy among the participants. Table 2 presents the means and SDs for each measure.

### Feature Selection for Multiple Linear Regression

Before performing the stepwise linear regression, we examined the correlations between the independent variables and the dependent variables. Among the independent variables, the primary UTAUT predictors (performance expectancy, effort expectancy, facilitating conditions, and social influence) were significantly correlated. Similarly, the subfactors of resistance to change (routine seeking, emotional reaction, short-term thinking, and cognitive rigidity) were found to be significantly correlated with each other. Routine seeking, emotional reactions, and short-term thinking were significantly correlated with low job control. The primary UTAUT predictors were significantly correlated with the dependent variable and behavioral intention, whereas gender displayed a marginal correlation, with a *P* value of .10. The correlation coefficients and *P* values for all variables are summarized in Tables3  4, respectively.

**Table 3.** Pearson correlation matrix examining relationships among Unified Theory of Acceptance and Use of Technology predictors (performance expectancy, effort expectancy, facilitating conditions, and social influence), resistance to change factors (routine seeking, emotional reaction, short-term thinking, and cognitive rigidity), and job stress factors (high job demand and low job control). Correlations were assessed for multicollinearity detection prior to regression modeling. Significant intercorrelations were observed among Unified Theory of Acceptance and Use of Technology predictors and among resistance to change subfactors.

	Performance expectancy	Effort expectancy	Facilitating condition	Social influence	Routine seeking	Emotional reaction	Short-term thinking	Cognitive rigidity	High job demand	Low job control
**Performance expectancy**										
Correlation coefficient	—^a^	0.25^b^	0.18^b^	0.58^b^	–0.05	0.01	0.01	–0.01	0.06	−0.01
95% CI	—	0.13 to 0.36	0.06 to 0.29	0.49 to 0.65	–0.18 to 0.07	–0.12 to 0.13	–0.11 to 0.13	–0.13 to 0.11	−0.06 to 0.18	−0.13 to 0.11
**Effort expectancy**										
Correlation coefficient	—	—	0.43^b^	0.40^b^	–0.04	–0.06	–0.03	0.08	−0.10	−0.02
95% CI	—	—	0.33 to 0.53	0.29 to 0.50	–0.16 to 0.08	–0.18 to 0.06	–0.15 to 0.09	–0.04 to 0.20	−0.22 to 0.02	−0.14 to 0.10
**Facilitating condition**										
Correlation coefficient	—	—	—	0.29^b^	–0.03	–0.06	–0.03	0.08	0.04	−0.04
95% CI	—	—	—	0.18 to 0.40	–0.15 to 0.10	–0.18 to 0.06	–0.15 to 0.10	–0.04 to 0.20	−0.08 to 0.16	−0.16 to 0.08
**Social influence**										
Correlation coefficient	—	—	—	—	0.04	0.05	0.09	0.09	0.08	0.04
95% CI	—	—	—	—	–0.08 to 0.16	–0.07 to 0.17	–0.03 to 0.21	–0.03 to 0.21	−0.04 to 0.20	−0.08 to 0.16
**Routine seeking**										
Correlation coefficient	—	—	—	—	—	0.54^b^	0.47^b^	0.38^b^	–0.08	0.20^b^
95% CI	—	—	—	—	—	0.45 to 0.62	0.37 to 0.56	0.27 to 0.48	−0.20 to 0.04	0.08 to 0.31
**Emotional reaction**										
Correlation coefficient	—	—	—	—	—	—	0.40^b^	0.26^b^	0.11	0.14^b^
95% CI	—	—	—	—	—	—	0.29 to 0.49	0.14 to 0.37	−0.01 to 0.23	0.02 to 0.26
**Short-term thinking**										
Correlation coefficient	—	—	—	—	—	—	—	0.30^b^	0.00	0.16^b^
95% CI	—	—	—	—	—	—	—	0.19 to 0.41	−0.12 to 0.12	0.04 to 0.28
**Cognitive rigidity**										
Correlation coefficient	—	—	—	—	—	—	—	—	−0.02	−0.01
95% CI	—	—	—	—	—	—	—	—	−0.14 to 0.11	−0.13 to 0.11
**High job demand**										
Correlation coefficient	—	—	—	—	—	—	—	—	—	0.22^b^
95% CI	—	—	—	—	—	—	—	—	—	0.10 to 0.33
**Low job control**										
Correlation coefficient	—	—	—	—	—	—	—	—	—	—
95% CI	—	—	—	—	—	—	—	—	—	—

a Not applicable.

b *P*<.05.

**Table 4.** Pearson correlations examining relationships between predictor variables (demographics: gender and age; Unified Theory of Acceptance and Use of Technology constructs: performance expectancy, effort expectancy, facilitating conditions, and social influence; resistance to change factors: routine seeking, emotional reaction, short-term thinking, and cognitive rigidity; job stress factors: high job demand and low job control) and outcome variable (behavioral intention to adopt virtual reality exposure therapy). Performance expectancy (*r*=.627) and social influence (*R*=.620) demonstrated the strongest correlations with adoption intention.

Variable	Correlation coefficient	95% CI	*P* value
Gender	0.103	–0.020 to 0.222	.10
Age	–0.025	–0.146 to 0.098	.69
Performance expectancy	0.627	0.547 to 0.696	<.01^a^
Effort expectancy	0.222	0.103 to 0.335	<.01^a^
Facilitating conditions	0.185	0.065 to 0.301	<.01^a^
Social influence	0.620	0.539 to 0.690	<.01^a^
Routine seeking	–0.038	–0.159 to 0.085	.55
Emotional reaction	–0.017	–0.139 to 0.105	.79
Short-term thinking	0.033	–0.089 to 0.155	.60
Cognitive rigidity	–0.011	–0.133 to 0.111	.86
Job high demand	0.063	–0.059 to 0.184	.31
Job low control	–0.014	–0.136 to 0.109	.83

a Significant at* P*<.05.

Forward selection using the predictors of performance expectancy, effort expectancy, facilitating conditions, and social influence identified performance expectancy and social influence as the strongest predictors of behavioral intention. The final model’s explanatory power was 0.494, with standardized coefficients *β* of .404 and .387 for performance expectancy (95% CI 0.297-0.512) and social influence (95% CI 0.280-0.494), respectively. Both confidence intervals were relatively narrow and did not include zero, indicating precise estimates and statistical significance. The detailed model statistics are presented in Table 5.

**Table 5.** Final regression model predicting behavioral intention to adopt virtual reality exposure therapy identified through forward stepwise selection from 4 Unified Theory of Acceptance and Use of Technology predictors (performance expectancy, effort expectancy, facilitating conditions, and social influence). Performance expectancy (*β*=.404, 95% CI 0.297-0.512, *P*<.001) and social influence (*β*=.387, 95% CI 0.280-0.494, *P*<.001) emerged as significant predictors, jointly explaining 49.4% of variance in adoption intention (*R*^2^=0.494). Model fit: *R*=.703, MSE=.512, *F*_2, 255_=124.3, *P*<.001. Narrow CIs indicate precise parameter estimation. All coefficients were estimated at 95% confidence level.

Outcome variable: behavioral intention	Unstandardized coefficients		Standardized coefficients, *β* (95% CI)	t	*P* value
	B	SE			
Constant^a^	1.936	0.674	—^b^	0	1
Performance expectancy	0.297	0.040	0.404 (0.297-0.512)	7.42	<.001
Social influence	0.407	0.057	0.387 (0.280-0.494)	7.10	<.001

a Predicted baseline value.

b Not applicable.

### Multiple Linear Regression With a Moderation Effect

**Figure 3.**
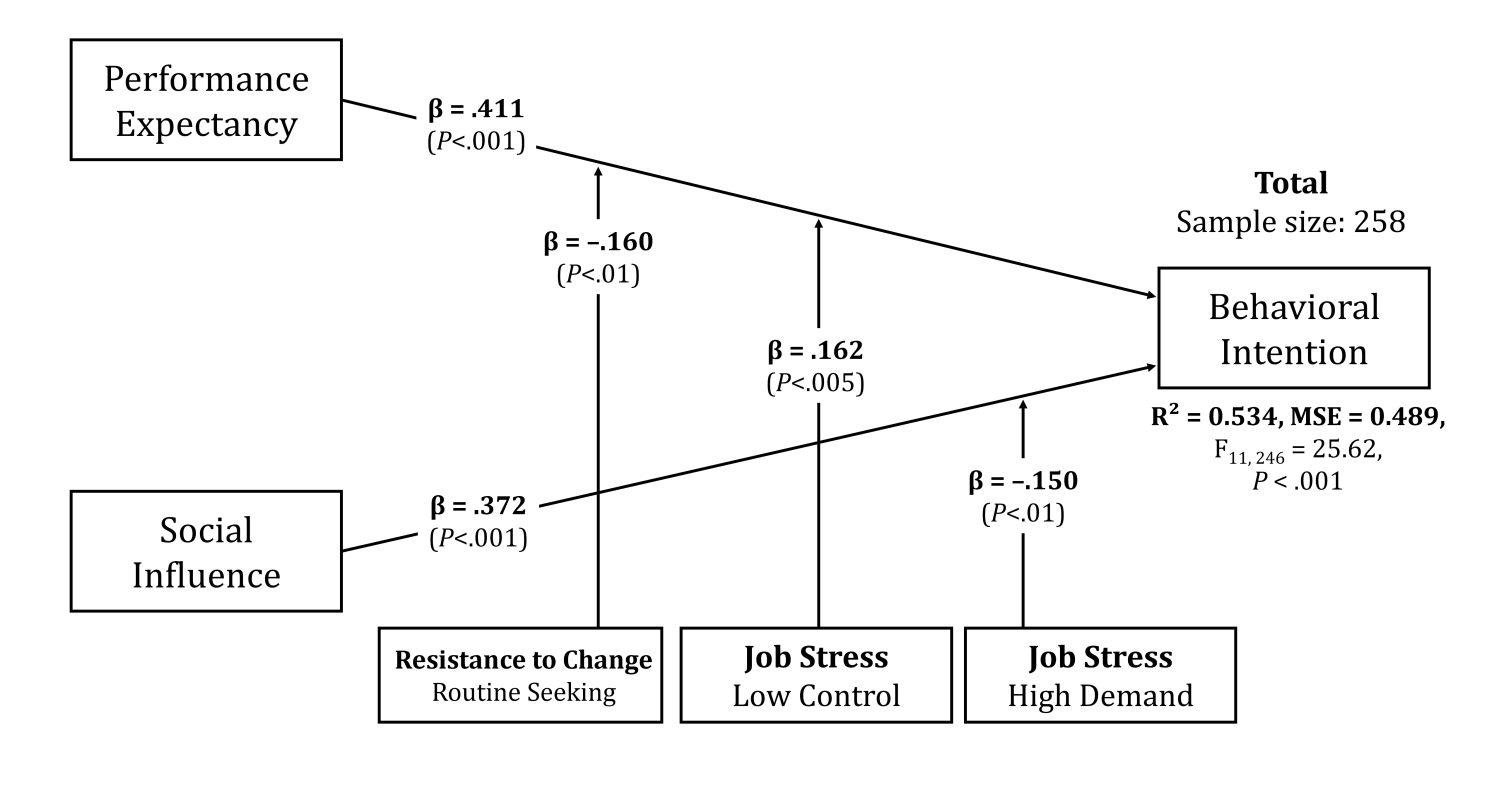
Path diagram showing final moderated regression model predicting VRET adoption intention for the full sample of Korean counselors (n=258). Main effects: performance expectancy (*β*=0.411, *P*<.001) and social influence (*β*=0.372, *P*<.001) predict behavioral intention (*R*^2^=0.534). Significant interaction effects: routine seeking × performance expectancy (*β*=−.160, *P*<.01), low job control ×performance expectancy (*β*=.162, *P*<.005), high job demand ×social influence (*β*=−.150, *P*=.01). Solid lines indicate statistically significant paths; dashed lines indicate nonsignificant paths. All coefficients are standardized.

The moderating effects of gender, age, subfactors of resistance to change (routine seeking, emotional reaction, short-term thinking, and cognitive rigidity), and subfactors of job stress (low job control and high job demands) were calculated. Among these, routine seeking, low job control, and high job demands demonstrated significant moderating effects and were retained in the final model. The final model revealed that routine seeking, low job control, and high job demand moderated the effect of performance expectancy (*β*=−.160, 95% CI –0.277 to −0.043), (*β*=.162, 95% CI 0.053-0.271), and social influence (*β*=−.150, 95% CI −0.26 to −0.036), respectively. These narrow intervals indicate precise estimation of the moderation effects, with the intervals excluding zero confirming their statistical significance. The *R*^2^ of the final model was 0.534, indicating an increase in explanatory power due to the moderating effects, with an MSE of .559 and *F*=25.62. The detailed values and final model are presented in Table 6 and Figure 3, respectively.

**Table 6.** Final moderated regression model testing whether resistance to change factors (routine seeking, emotional reaction, short-term thinking, and cognitive rigidity) and job stress factors (high job demand and low job control) moderate relationships between Unified Theory of Acceptance and Use of Technology predictors (performance expectancy and social influence) and virtual reality exposure therapy adoption intention. Main effects: performance expectancy (*β*=.411, *P*<.001) and social influence (*β*=.372, *P*<.001) remained significant predictors. Significant interaction effects: routine seeking×performance expectancy (*β*=–.160, *P*<.01), low job control×performance expectancy (*β*=.162, *P*<.005), and high job demand×social influence (*β*=–.150, *P*=.01). Model fit: *R*^2^=0.534 (53.4% variance explained), *F*_11, 246_=25.62, *P*<.001. All coefficients estimated at 95% confidence level.

Outcome variable: behavioral intention	Unstandardized coefficients		Standardized coefficients, *β* (95% CI)	t	*P* value
	B	SE			
Constant^a^	–4.873	4.178	–^b^	0.12	.90
Performance expectancy	0.293	0.226	.411 (0.303 to 0.519)	7.51	<.001
Social influence	0.101	0.328	.372 (0.265 to 0.480)	6.82	<.001
Routine seeking	0.536	0.237	–.026 (–0.115 to 0.062)	–0.59	.56
Job high demand	0.308	0.157	.005 (–0.085 to 0.094)	0.10	.92
Job low control	–1.298	0.585	–.004 (–0.095 to 0.088)	–0.08	.94
Performance expectancy: routine seeking	–0.039	0.014	–.160 (–0.277 to –0.043)	–2.70	<.01
Performance expectancy: high job demand	0.002	0.009	.011 (–0.103 to 0.125)	0.19	.85
Performance expectancy: low job control	0.098	0.034	.162 (0.053 to 0.271)	2.93	<.005
Social influence: routine seeking	0.022	0.019	.064 (–0.041 to 0.169)	1.19	.23
Social influence: high job demand	–0.033	0.013	–.150 (–0.264 to –0.036)	–2.60	.01
Social influence: low job control	–0.067	0.045	–.076 (–0.178 to 0.026)	–1.48	.14

a Predicted baseline value.

b Not applicable.

### Moderation Effects by Age Group

#### Overview

To further examine the moderating effects of age, the participants were divided into 3 age groups. Age ranges were determined based on a mean age of 42.17 years and half of the SD (4.42 y, SD 8.84). This resulted in the following groups: young (37 years or younger, n=91), middle (38‐47 y, n=87), and old (48 years or older, n=80). Due to the low percentage of male participants in the overall sample (34/258 participants, 13.2%) and the reduced number of males in each age group, gender was excluded as a moderator. Instead, the moderating effects of routine seeking, a subfactor of resistance to change, low job control, high job demands, job stress, and age were reevaluated.

#### Young Group (37 Years or Younger)

**Figure 4.**
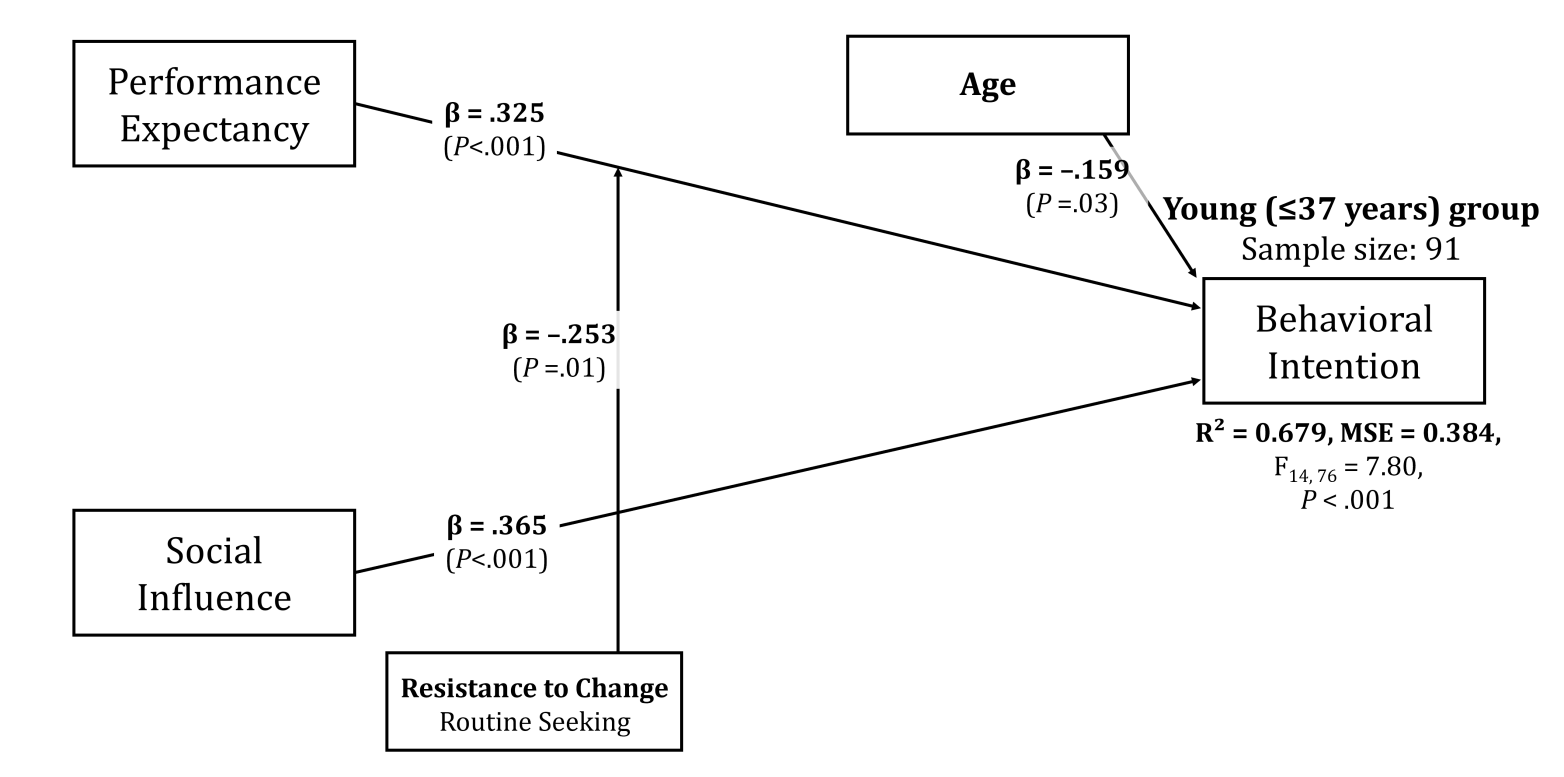
Path diagram for young counselor subgroup (≤37 y, n=91) showing distinct moderation patterns. Performance expectancy (*β*=.325, *P*<.001) and social influence (*β*=.365, *P*<.001) predict adoption intention (*R*^2^=0.679). Routine seeking significantly moderates performance expectancy (*β*=−.253, *P*<.05), and age shows a direct negative effect on behavioral intention (*β*=−.159, *P*<.05). Young counselors demonstrated greater sensitivity to individual resistance factors compared with other age groups. Solid lines indicate significant paths; standardized coefficients are shown.

In this group, more diverse moderation effects were observed than in the full sample. The main effect of performance expectancy was slightly lower than that of the full sample (*β*=.325, 95% CI 0.131-0.520), whereas the main effect of social influence remained similar (*β*=.365, 95% CI 0.193-0.536). The wider confidence intervals in this subgroup reflect reduced precision due to a smaller sample size, but both effects remain statistically significant. The explanatory power increased to 0.679. Routine seeking, a subfactor of resistance to change, was positively related to performance expectancy (*β*=−0.253, 95% CI −0.452 to −0.054). Age showed no moderation effect but had a direct effect on behavioral intention (*β*=−.159, 95% CI −0.303 to −0.015), though the confidence interval approaching zero suggests this effect should be interpreted cautiously. These results suggest that younger counselors are more likely to adopt VRET when they exhibit lower routine [1] tendencies. Additionally, the impact of performance expectancy on adoption intention was more substantial when routine seeking was low. The results for this group are shown in Figure 4.

#### Middle Group (38‐47 Years)

**Figure 5.**
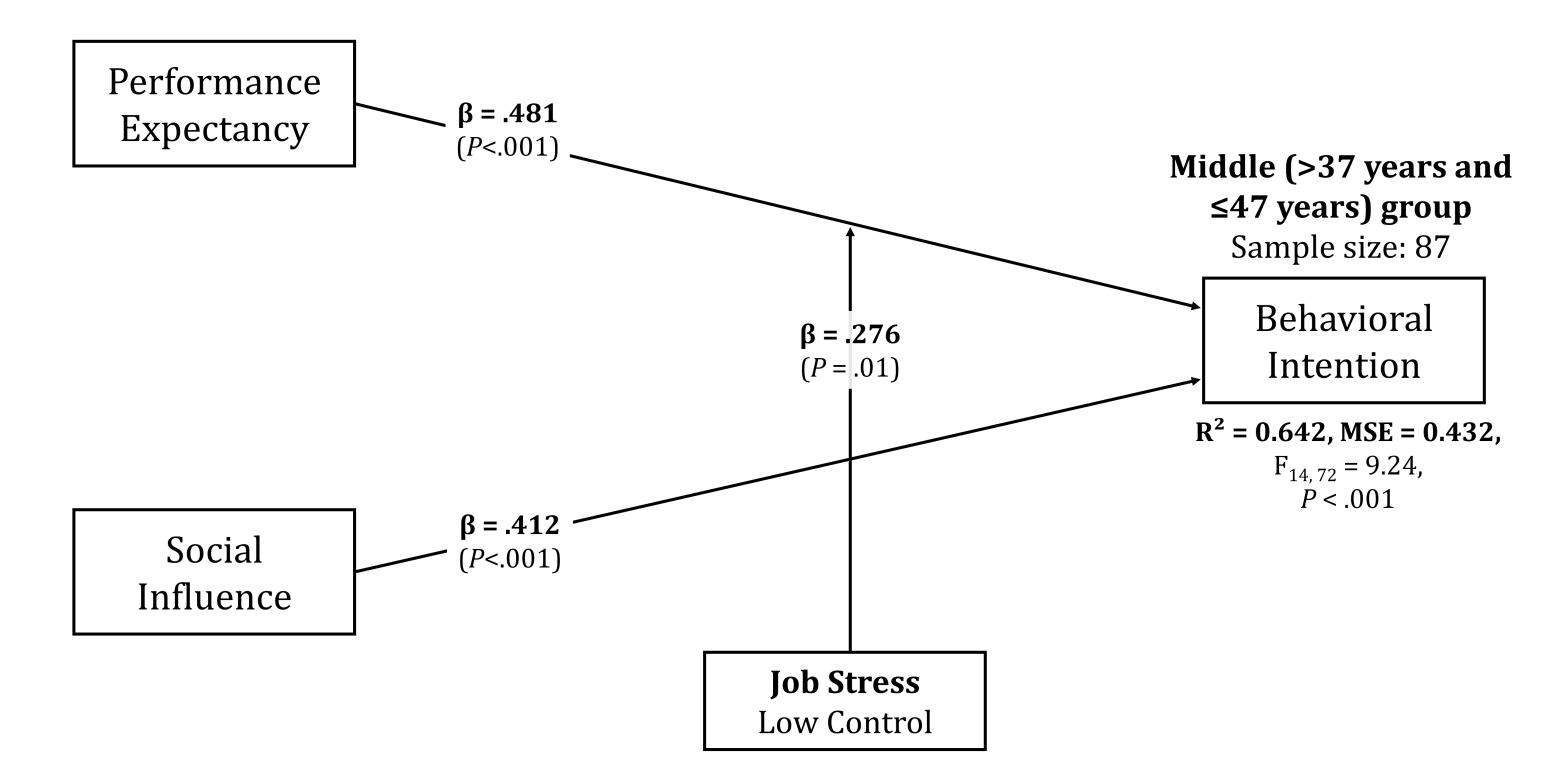
Path diagram for middle-aged counselor subgroup (38‐47 y, n=87) showing work context effects. Performance expectancy (*β*=0.481, *P*<.001) and social influence (*β*=0.412, *P*<.001) show stronger effects than the full sample (Figure 3). Low job control significantly moderates performance expectancy (*β*=0.276, *P*<.05), indicating workplace autonomy amplifies performance expectations’ influence on adoption intention (*R*^2^=0.642). Unlike young counselors (Figure 4), resistance to change factors show no significant moderation. Solid lines indicate significant paths; standardized coefficients are shown.

In the middle group, the main effects of performance expectancy (*β*=.481, 95% CI 0.291-0.671) and social influence (*β*=.412, 95% CI 0.227-0.597) were higher than the full-sample results. The confidence intervals, while broader than those for the full sample, indicate reasonably precise estimates for both predictors. The explanatory power of the model increased to 0.642. No moderation effect of routine seeking was found, but low job control exhibited an increased moderation effect on performance expectancy (*β*=.276, 95% CI 0.080-0.473). The confidence interval for this moderation effect, with its lower bound close to zero, suggests this relationship should be interpreted with some caution. These findings suggest that although routine seeking does not play a role, low job control may reduce adoption intention, and the impact of performance expectancy on adoption intention was relatively more substantial in the middle group. The final results for this group are shown in Figure 5.

#### Old Group (48 Years or Older)

**Figure 6.**
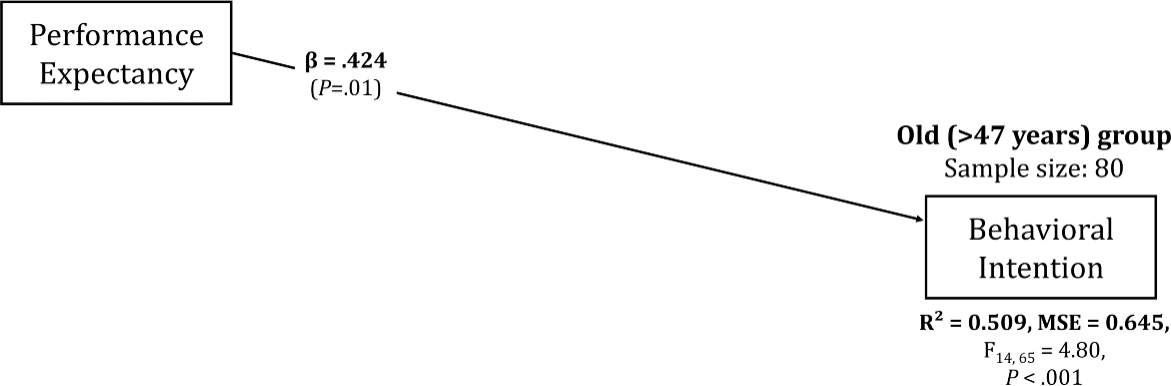
Path diagram for older counselor subgroup (≥48 y, n=80) showing simplified adoption pattern. Performance expectancy remains a strong predictor (*β*=0.424, *P*<.001), while social influence shows only marginal significance (*β*=0.262, *P*=.09). No significant moderating effects were observed for resistance to change or job stress factors (*R*^2^=0.509). This pattern contrasts with younger groups (Figures 4 and 5) where contextual and individual factors significantly moderated adoption predictors, suggesting older counselors rely primarily on perceived effectiveness for adoption decisions. Solid lines indicate significant paths; dashed line indicates marginal significance; standardized coefficients are shown.

No significant moderating effects were observed in the old group. The main effect of performance expectancy increased (*β*=.424, 95% CI 0.135-0.713), whereas the main effect of social influence decreased (*β*=.262, 95% CI −0.043-0.566), with its significance level increasing to marginal significance (*P*=.09). The very wide confidence intervals in this group reflect the reduced precision due to the smaller sample size and greater variability in responses. Notably, the confidence interval for social influence includes zero, explaining its marginal significance. The explanatory power of the model decreased slightly to 0.509. The diminished significance of the social influence’s main effect suggests that performance expectancy had a significant influence on adoption intention than social influence for older counselors. Other factors such as age, job stress, and resistance to change did not significantly influence adoption intention in this group. The results for this group are shown in Figure 6.

These results highlight the variability in moderation effects across age groups, suggesting that younger and middle-aged counselors may respond more to contextual and psychological factors (eg, job stress and resistance to change), whereas older counselors prioritize performance expectancy when considering the adoption of VRET.

### Thematic Results

#### Overview

The thematic analysis encompassed 290 meaning units derived from 257 open-ended responses, as one participant did not provide answers, while others articulated multiple ideas within a single response. Through the iterative coding process, 31 initial codes were identified. These codes were organized into 3 major themes and 9 subthemes, with frequency counts ranging from 11 to 103 mentions across the sample. The analysis prioritized the identification of implementation factors and contextual considerations that extended beyond the quantitative UTAUT findings, with a particular focus on practical barriers, facilitators, and professional concerns not fully captured in the quantitative predictors. The complete thematic structure is presented in Table 7.

**Table 7.** Thematic structure identified through team-based consensus coding of open-ended survey responses regarding virtual reality exposure therapy (VRET) applicability and improvement needs (257 respondents providing 290 meaning units). Three major themes and 10 subthemes emerged: (1) Counselor evaluation criteria for VRET (content diversity, scientific validation, integration with existing therapy, and preparatory practice utility), (2) Considerations for promoting VRET to counselors (technology resistance, targeted promotion needs, and ongoing development requirements), (3) Continuous improvement areas for field implementation (counselor training, customizable options, and clear guidelines). Themes focus on implementation factors extending beyond the Unified Theory of Acceptance and Use of Technology constructs captured quantitatively. Responses aligning with the Unified Theory of Acceptance and Use of Technology predictors (performance expectancy: 83 mentions; effort expectancy: 45 mentions; facilitating conditions: 79 mentions; social influence: 55 mentions) were recorded but excluded from thematic elaboration to emphasize novel insights complementing quantitative findings.

Theme	Subtheme
Counselor evaluation criteria for VRET	1‐1. A wide range of content types and levels should be available to meet clients’ needs. 1‐2. Scientific evidence on the effectiveness and safety of VR^a^ therapy is needed. 1‐3. Requires training or interaction that can be implemented alongside VR exposure. 1‐4. VR exposure therapy can serve as a practice for real exposure therapy.
Considerations for promoting and introducing VRET to counselors	2‐1. Existing resistance to unfamiliar technologies like virtual reality. 2‐2. The need for targeted promotion to increase awareness of VR exposure therapy. 2‐3. Essentiality of ongoing content development, distribution, and training.
Areas requiring continuous improvement for VRET field implementation	3‐1. Counselor training and pre-experience are required for effective delivery of VR exposure therapy. 3‐2. Customizable options for clients’ conditions and symptoms should be available in safe, controlled settings. 3‐3. Clear guidelines, manuals, and instructions are essential for VR use in counseling.

a VR: virtual reality.

#### Counselor Evaluation Criteria for VRET

The most prominent theme that emerged from the analysis was the counselors’ evaluation criteria when considering VRET adoption. Counselors assessed VRET based on specific standards related to content diversity, scientific validation, integration capabilities, and preparatory utility. Frequently mentioned subthemes included the need for a diverse array of content types customized to clients’ symptoms or situations (Subtheme 1‐1) and the requirement for scientific validation of VR’s efficacy and safety (Subtheme 1‐2).

*It would be beneficial to have content that varies according to specific situations and levels, such as those for panic disorder.*

[P29]

*Distinct evidence of effectiveness compared to other treatments is needed.*

[P176]

Beyond basic requirements, counselors emphasized VRET’s potential for integration with existing therapeutic approaches (Subtheme 1‐3) and its utility as preparatory practice before real-world exposure (Subtheme 1‐4).

*It would be better if VR exposure therapy included techniques such as assertion training or mindfulness for anxiety relief.*

[P44]

*VR content could be useful as a simulation practice for clients before they transition to experiences with counselors or others.*

[P55]

#### Considerations for Promoting and Introducing VRET to Counselors

The second major theme focused on considerations for promoting and introducing VRET to counselors, addressing both implementation challenges and facilitating strategies. As anticipated, some counselors expressed resistance to VR because of their unfamiliarity with it (Subtheme 2‐1).

*The concept of a virtual world evokes a lot of resistance. Reducing this resistance should be prioritized before implementing VR therapy.*

[P114]

However, this resistance was not overwhelming, with many counselors emphasizing the importance of ongoing content development, distribution, and promotion to render VRET more accessible and relatable in counseling practice (Subthemes 2‐2 and 2‐3).

*Promotion for professionals and the general public should be well–executed.*

[P19]

*It would be great if the content could be continually updated with various scenarios.*

[P130]

#### Areas Requiring Continuous Improvement for VRET Field Implementation

The third theme identified areas requiring continuous improvement for successful VRET field implementation, emphasizing the need for counselors to possess professional competence and for the system to be reliable and effective. They noted the need for intuitively manageable content, allowing counselors to become adept at its use (Subtheme 3‐1), while maintaining authority and control over the therapeutic process (Subtheme 3‐2). Additionally, they emphasized the necessity for structured training and guidance to ensure effective use (Subtheme 3‐3).

*Mastery in applying VR content and professionalism in effectively using it with clients are essential.*

[P207]

*It would be helpful to adjust settings such as characters, age, scenarios, and dialogues to replicate real-life situations in VR.*

[P130]

*Structured manuals or training should be provided to maintain quality control in VR therapy use.*

[P238]

## Discussion

### Principal Findings

#### Overview

This study examined the factors influencing Korean counselors’ acceptance of VRET within the UTAUT framework using a mixed methods approach. Our analysis aimed to (1) quantitatively identify key predictors through stepwise regression analysis, (2) examine moderating effects of individual characteristics and work environment factors, (3) explore age-related differences in acceptance patterns, and (4) provide contextual understanding through thematic analysis of counselors’ perspectives. The quantitative findings revealed that performance expectancy and social influence were the primary predictors of VRET acceptance intention, with significant moderating effects from routine seeking (resistance to change), job stress factors, and notable variations across age groups. The thematic analysis identified 3 major themes: counselors’ ambivalence toward VRET as either a treatment or supplementary tool, barriers and facilitators for introducing VR in counseling practice, and the need for seamless counselor control over VR technology. Together, these findings provide comprehensive insights for implementing VRET in Korean counseling practice and highlight the importance of addressing both technological capabilities and professional contextual factors in technology adoption strategies.

#### Age-Related Perspectives on New Technology

The major factors influencing the counselors’ adoption of VRET were primarily performance expectancy and social influence. Age, routine seeking, low job control, and high job demands moderated the effects of performance expectancy and social influence in the young group, indicating that individual characteristics and work environment significantly influenced adoption. Individual factors such as routine seeking and age did not serve as moderators in the middle group, leaving job-related factors with significant moderation effects. All moderation effects vanished, and the main effect of social influence became marginal, with performance expectancy as the dominant predictor in the old group. This trend may reflect the unique nature of counseling as a profession, in which younger counselors typically work under the supervision of experienced professionals and receive consistent guidance. For younger counselors, the lack of personal experience and the need to report to or be guided by superiors may lead to a greater sensitivity to external factors when adopting new tools such as VRET. Conversely, as experienced supervisors, older counselors may have confidence and objectivity in using new technologies to enhance performance without being influenced by external factors. Notably, effort expectancy and facilitating conditions, despite showing significant correlations with behavioral intention (Table 4), did not emerge as predictors in the stepwise regression. This pattern aligns with similar findings in mental health technology adoption studies. An extended UTAUT study of e–mental health service acceptance among German and Swiss psychotherapists found that effort expectancy and facilitating conditions were not significant predictors, while performance expectancy and context-specific barriers and advantages emerged as primary factors [46]. In a study examining mental health practitioners’ acceptance of virtual humans, effort expectancy showed only a weak significant effect, with researchers explaining that society’s standardized use of new technologies over the past decade has reduced fears about technological complexity and usage rejection [47]. Facilitating conditions were nonsignificant in the same study, primarily attributed to the overall lack of VR equipment availability in Spanish hospitals [47]. Similarly, our study context reflects a comparable infrastructure limitation, as Korean counseling centers currently lack established VR equipment and systems, which may explain why facilitating conditions did not emerge as a significant predictor despite counselors’ theoretical interest in VRET adoption.

This result is consistent with the original UTAUT framework, which posits that age and experience can moderate the effects of major constructs: older individuals tend to be more influenced by ease of use, social influence, and facilitating conditions, whereas younger individuals respond positively to performance expectancy [48]. Although the direct effect of psychotherapists’ age on VR acceptance has been found in previous studies, albeit minimally [28], this study analyzed the differences between each age group as a moderating effect for the first time. These results followed this trend; older or more experienced counselors in the sample seemed to be less swayed by social influence or perceived pressure, potentially owing to greater professional confidence, yet they would be open to VRET if it were simple to use and advantageous. Younger counselors were generally more receptive to new technology and more influenced by external opinions or organizational support that endorsed VRET, reflecting a higher sensitivity to social influence (eg, the desire to use tools favored by supervisors or colleagues) [49]. Our finding that social influence significantly predicted VRET adoption aligns with collectivistic cultural patterns [50], contrasting with German therapist studies where performance expectancy and facilitating conditions were primary predictors. This difference may reflect Korean emphasis on social consensus in professional decision-making compared with more individualistic Western approaches.

#### Supporting Role of VRET in Counseling Practice

The thematic analysis revealed that counselors evaluate VRET based on specific criteria (Theme 1) and prefer it as a supplementary tool to enhance counseling effectiveness through exposure practice, consistent with findings on practical requirements (Theme 3). Counselors expressed a preference for proven tools but did not want to be reliant on them. This decision implies that detailed implementation environments, specific target groups, or complex operational procedures may hinder VRET adoption in real-world counseling contexts, even if such setups are effective in controlled laboratory environments. These concerns were particularly pronounced among counselors who experienced high job demands and low job control, reflecting the concept of technostress identified in previous research. When counselors are overwhelmed by their workload or lack autonomy in selecting therapeutic tools, they may not have the time or psychological resources to engage with new technology, resulting in greater resistance to its adoption [51].

#### Practical Implementation of VRET

For VRET to be successfully implemented in the real world, it is essential to promote the technology and develop accessible and diverse VRET applications in both academic and industrial contexts. Although VR has demonstrated outstanding performance and efficacy in exposure therapy, it may be unfamiliar or even intimidating to clients with anxiety or panic disorders and counselors who empathize with and care for them [52]. Even if VRET is not immediately adopted, awareness of ongoing advancements in VRET research, as well as national projects supporting its development and validation, could foster acceptance in academic and counseling communities. Additionally, national support is needed to provide VR-based experiences and exposure therapy programs in trusted counseling and mental health welfare centers. This method would help clients, the general public, and professionals, such as doctors and counselors, become familiar with VR content, thereby facilitating efficient adaptation to emerging technology.

### Strengths

This study makes several novel contributions that advance VRET acceptance research beyond existing literature. First, we examined certified counselors working across diverse nonhospital settings—universities, public institutions, corporate counseling centers, and private practices. Unlike previous studies that predominantly focused on physicians in hospital environments [19,53], this expansion addresses a critical gap as VRET technology becomes increasingly accessible beyond traditional clinical settings. Counselors serve as frontline providers of exposure-based interventions, yet their acceptance patterns remain largely understudied despite their essential role in mental health service delivery.

Second, we extended the UTAUT framework by incorporating profession-specific moderators that existing studies have largely overlooked. Prior research has addressed organizational barriers to implementing evidence-based treatments [24,25] and the role of VR technology in mental health practice [26], yet systematic integration of occupation-specific psychological factors—such as resistance to change and job stress—as moderators within the UTAUT model remains limited. By including these factors alongside traditional demographic variables, we captured unique professional characteristics of counseling practice that influence technology adoption. This extension revealed that performance expectancy and social influence operate differently across age groups, reflecting hierarchical organizational structures rather than purely individual decision-making processes—a dimension not adequately addressed in prior research [54,55].

Third, our age-stratified analysis uncovered culturally-specific adoption mechanisms not captured in predominantly Western samples. Younger counselors showed greater sensitivity to social influence and individual characteristics, middle-aged counselors were primarily influenced by job autonomy, while older counselors relied predominantly on performance expectancy. This pattern reflects the hierarchical nature of Korean counseling organizations, where age correlates strongly with professional roles and decision-making authority, diverging from individualistic Western contexts where technology adoption is conceptualized as individual-level decisions [46,47]. These findings highlight the importance of cultural and organizational context in TAMs, suggesting that implementation strategies must be adapted to collectivistic professional cultures.

Methodologically, we used a convergent mixed methods approach combining predictive modeling with thematic analysis to provide both statistical generalizability and contextual depth. Importantly, we deliberately excluded UTAUT-related items during thematic analysis—contrary to previous qualitative VR studies that categorized themes within existing UTAUT predictors. This approach revealed implementation considerations not fully captured by quantitative measures: counselors’ nuanced views of VRET as a supplementary rather than replacement tool, their emphasis on maintaining therapeutic control while leveraging technological capabilities, and specific content requirements for different anxiety presentations.

Practically, our findings provide immediately actionable guidance as VRET technology advances and costs decline with standalone devices [13,56]. The identification of performance expectancy and social influence as primary predictors, moderated by resistance to change and job stress, directly informs implementation strategies: (1) provide robust effectiveness evidence through demonstration projects, (2) leverage professional networks and endorsements from respected colleagues, (3) address workflow integration barriers particularly for counselors with high job demands, and (4) develop age- and seniority-stratified dissemination strategies recognizing hierarchical organizational structures. These insights are applicable to VRET content developers designing Korean-language exposure scenarios, policymakers planning technology implementation initiatives beyond hospital settings, and researchers designing future effectiveness trials.

Finally, our findings contribute to health technology acceptance literature in collectivistic cultures [57]. The prominence of social influence in predicting Korean counselor adoption—comparable to performance expectancy—contrasts with Western studies where performance expectancy and facilitating conditions often dominate [46]. This cultural specificity underscores that technology dissemination strategies must account for cultural values, organizational hierarchies, and professional decision-making norms characteristic of specific national contexts.

### Limitations

The limitation of this study is that the participants likely had varying levels of experience and familiarity with VR. As VR is a relatively novel technology for most individuals, its use can significantly influence its adoption [20]. To address this, a 4-minute introductory video was created to provide information on VRET’s purpose, evidence, content, and use. However, as the survey was conducted via the web, it was unclear whether all participants had watched the videos. Future studies should verify video viewing and investigate the type and extent of prior VR use among participants. Additionally, we did not assess participants’ therapeutic orientations (eg, CBT, psychodynamic, and humanistic), which could significantly influence VRET acceptance as exposure-based interventions align more closely with cognitive-behavioral approaches than with other therapeutic modalities [58]. Self-report bias may have inflated acceptance ratings and could explain why some expected predictors (effort expectancy and facilitating conditions) did not emerge as significant, as participants may have underestimated the implementation challenges they had not experienced.

Another limitation is that the thematic analysis relied on open-ended survey responses rather than interviews. Although this allowed for the aggregation of a larger number of perspectives, interpreting the intended meaning of the textual responses was sometimes challenging, leading to inevitable data loss. Nevertheless, by not conducting interviews, the study was able to collect diverse opinions. It included mention frequencies in the thematic analysis, which is more comprehensive than traditional interview-based qualitative studies. The cultural specificity of our Korean sample, characterized by hierarchical structures and collectivistic decision-making, may limit generalizability to individualistic cultures where different UTAUT predictors might be more salient [59].

### Conclusions

Counselors in South Korea expressed positive attitudes toward adopting VRET, with adoption primarily driven by performance expectancy and social influence. This study addresses critical gaps in technology acceptance research by examining certified counselors working across universities, public institutions, and private clinics—settings previously neglected in VRET literature that predominantly focused on hospital-based physicians [19,53]. As VRET technology becomes increasingly accessible through affordable standalone devices, understanding acceptance patterns beyond traditional clinical environments is essential for broader implementation.

This study extends the UTAUT framework by integrating profession-specific psychological and occupational factors—resistance to change and job stress—that significantly moderated technology acceptance effects. Routine seeking weakened performance expectancy’s influence, while job control and job demands altered how counselors responded to perceived effectiveness and social pressures. Age-stratified analysis revealed distinct adoption patterns reflecting hierarchical organizational structures in Korean counseling practice: younger counselors showed greater sensitivity to social influence and individual resistance factors, middle-aged counselors prioritized work autonomy, while older counselors relied predominantly on performance expectancy. These patterns suggest technology dissemination strategies must account for organizational hierarchies and career stage differences rather than treating adoption as purely individual decisions.

Our mixed methods approach revealed implementation requirements extending beyond UTAUT’s traditional focus on usability and facilitating conditions. Thematic analysis demonstrated counselors evaluate VRET through specific professional criteria—scientific validation, content diversity, therapeutic control—and prefer it as a supplementary tool rather than a replacement technology. Practical implementation requires disseminating robust effectiveness evidence, leveraging professional networks for peer endorsement, addressing work environment barriers particularly for high-demand contexts, developing diverse culturally-adapted content, providing structured training programs, and ensuring clear usage guidelines with institutional support. These insights directly inform VRET content developers designing Korean scenarios, policymakers planning technology implementation initiatives beyond hospital settings, and researchers designing effectiveness trials in hierarchical professional contexts.

## Supplementary material

10.2196/81803 Multimedia Appendix 1 Four-minute Korean-language educational video about virtual reality exposure therapy shown to all participants (n=258) before survey completion.

10.2196/81803 Checklist 1 Gramms checklist summarizing the mixed methods rationale, integration, sampling, analysis, and limitations for this study.

10.2196/81803 Checklist 2 COREQ 32-item checklist for the thematic analysis of open-ended responses in this study.

## Abbreviations

COREQ: Consolidated Criteria for Reporting Qualitative Research
GRAMMS: Good Reporting of A Mixed Methods Study
TAM: Technology Acceptance Model
UTAUT: Unified Theory of Acceptance and Use of Technology
VR: virtual reality
VRET: virtual reality exposure therapy
