# Supplementary material for: Exploring Predictors of Counselors’ Acceptance of Virtual Reality Exposure Therapy With Resistance and Job Contexts as Moderators: Cross-Sectional Mixed Methods Study
Source: J Med Internet Res. 2025 Dec 31;27:e81803. doi: 10.2196/81803 (PMC12755899; doi:10.2196/81803)
Supplement: Checklist 1 [file jmir-v27-e81803-s002.docx]

Table S1. GRAMMS Checklist

| Item | How it was addressed | Supporting quotes from the manuscript | Location in manuscript |
| --- | --- | --- | --- |
| 1. Rationale for using mixed methods | This study combined quantitative analysis based on the UTAUT model with qualitative exploration through open-ended questions. This design aimed to achieve a multidimensional understanding of counselors’ acceptance of VRET. | “Objective: … We used the Unified Theory of Acceptance and Use of Technology (UTAUT) as a base model, but added extra factors … We also asked open-ended questions to understand what counselors thought about VRET more deeply.” / “This study examined … (1) quantitatively identified key predictors … (4) conducted a thematic analysis to gain contextual understanding…” | Abstract; Introduction (Study Aim) |
| 2. Study design (purpose, priority, sequence) | The purpose of the design was complementary and expansionary, with quantitative methods prioritized for addressing primary research questions while qualitative data provided contextual understanding. Data were collected simultaneously within the same survey instrument using a convergent approach. | “This study employed convergent mixed-methods, cross-sectional design, with quantitative methods prioritized to address the primary research questions, while qualitative data provided complementary and expansionary insights. In line with the convergent mixed-methods design, both quantitative and qualitative data were collected remotely and simultaneously within a single survey instrument that integrated closed and open-ended items.” | Methods – Study setting and design |
| 3. Sampling, data collection, and analysis | Quantitative data were collected from 258 nationally certified counselors across Korea via email recruitment, with prior sample size estimation using G*Power. Analyses included stepwise multiple regression and moderation analysis using Google Colab and sklearn. Qualitative data were obtained through open-ended survey responses and analyzed in QDA Miner. Three researchers followed a five-step consensus-based coding process: (1) segmenting responses into meaning units, (2) developing preliminary categories, (3) iterative coding with codebook updates, (4) finalizing themes with definitions and representative quotes, and (5) applying a 4% frequency threshold for theme retention. | “For open-ended responses, thematic analysis was performed using the qualitative data analysis tool QDA Miner. The open-ended questions explored participants’ general perceptions of VR exposure therapy content and potential improvements for content targeting panic and social anxiety. Unlike traditional interview–based studies that pursue sequential saturation, our predetermined large sample (n=258) provided comprehensive perspective coverage, significantly exceeding the typical qualitative sample sizes of 6–12 interviews [42]. Sample adequacy was established through systematic consensus–building among multiple coders and frequency–based validation criteria.  …  The thematic analysis aimed to identify factors not fully captured in the quantitative UTAUT analysis, particularly contextual interpretations of effort expectancy and facilitating conditions, and to provide a deeper understanding of the quantitative findings. Codes directly reflecting quantitative results (e.g., performance expectancy predicting adoption intention) were minimally incorporated into higher–level themes to avoid redundancy and focus on novel insights complementing the quantitative analysis. | Methods –Thematic Analysis |
| 4. Integration (timing, approach, personnel) | Integration occurred during data collection (same survey instrument) and interpretation phases. Three researchers conducted both quantitative coding and qualitative analysis. Integration involved comparing qualitative themes with quantitative predictors while excluding UTAUT-related themes to avoid redundancy. | "This study employed a cross–sectional, mixed–methods design combining quantitative survey data with qualitative thematic analysis of open–ended responses." / "this study excluded UTAUT–related items during thematic analysis, resulting in more specific and practical insights." | Methods – Study setting and  Discussion – Strengths |
| 5. Limitations due to mixed methods | Limitations included: (1) inability to confirm whether all participants attentively watched the online video, (2) qualitative data were based only on open-ended responses rather than interviews, leading to potential ambiguity or data loss, (3) lack of assessment of counselors’ therapeutic orientation, which could affect acceptance, and (4) cultural specificity of the Korean sample, limiting generalizability. Excluding UTAUT-related codes in the qualitative analysis was presented as a methodological strategy to avoid redundancy and derive novel insights. | “The limitation of this study is that … it was unclear whether all participants had watched the videos.” / “Another limitation is that the thematic analysis relied on open–ended survey responses rather than interviews … leading to inevitable data loss.” / “We did not assess participants’ therapeutic orientations …” / “The cultural specificity of our Korean sample … may limit generalizability …” | Discussion – Limitations |
| 6. Insights gained from mixed methods | The mixed-methods approach provided insights not obtainable through a single method. • Quantitative: Performance expectancy and social influence significantly predicted adoption intention (R² = .494). Moderation effects varied by age (e.g., younger counselors were more sensitive to contextual factors, older counselors emphasized performance expectancy). • Qualitative: Counselors identified evaluation criteria (diverse content, scientific validation, integrative applicability), promotion and introduction considerations (resistance, need for continuous updates), and areas for practical improvement (professional competence, training, reliable systems). Together, these findings extended explanatory power and offered concrete implementation strategies. | “Results: Performance expectancy … and social influence … predicted VRET adoption intentions (R² = .494). Younger counselors were more sensitive … older counselors prioritized performance expectancy.” / “Thematic analysis identified three themes: (1) Counselor evaluation criteria for VRET … (2) Considerations for promoting and introducing VRET … (3) Areas requiring continuous improvement …” / “Counselors primarily considered whether VRET could serve as a supportive tool … continuous and positive promotion … diverse content … is necessary.” | Results; Discussion; Conclusion |
