# Supplementary material for: Exploring Predictors of Counselors’ Acceptance of Virtual Reality Exposure Therapy With Resistance and Job Contexts as Moderators: Cross-Sectional Mixed Methods Study
Source: J Med Internet Res. 2025 Dec 31;27:e81803. doi: 10.2196/81803 (PMC12755899; doi:10.2196/81803)
Supplement: Checklist 2 [file jmir-v27-e81803-s003.docx]

Table S2. COREQ Checklist

| Domain | Item | As Applied in This Study | Location in Manuscript |
| --- | --- | --- | --- |
| Domain 1. Research team and reflexivity | | | |
| Personal characteristics | 1. Interviewer/facilitator | Not applicable (open-ended online survey responses only) | Methods – Data collection |
|  | 2. Credentials | First author (MK): BS, fourth-year graduate student; Second author (MJ): BSN, second-year graduate student; Third author (YL): BS, first-year graduate student. (These three authors are in the combinded course for master's and doctoral degree.) | Title page, Affiliations; Methods – Thematic analysis |
|  | 3. Occupation | Graduate student researchers | Title page |
|  | 4. Gender | Coders: 1 male, 2 female | Not reported (N/R) in manuscript |
|  | 5. Experience/training | Team-based consensus approach. First author had prior qualitative research experience; second and third authors trained via textbooks and exemplar studies | Methods – Thematic analysis |
| Relationship | 6. Relationship established | No prior personal relationship; participants recruited via professional association email | Methods – Recruitment |
|  | 7. Participant knowledge of interviewer | Participants were informed of study purpose, procedures, and ethics; consent obtained | Methods – Ethics |
|  | 8. Researcher characteristics (bias, assumptions, etc.) | No explicit reflexivity statement | N/R |
| Domain 2. Study design | | | |
| Theoretical framework | 9. Methodological orientation | Inductive team-based consensus thematic analysis with iterative codebook updates; ≥4% frequency threshold; QDA Miner used | Methods – Thematic analysis; Discussion – Strengths |
| Participant selection | 10. Sampling | Convenience sampling: invitation email sent to nationally certified counselors | Methods – Recruitment |
|  | 11. Method of approach | Email invitations sent through professional associations; Google Forms for data collection with informed consent | Methods – Recruitment |
|  | 12. Sample size | 258 participants, no exclusions for survey. | Results – Demographics |
|  | 13. Non-participation | Although there were no exclusions among the 258 participants (i.e., no data were arbitrarily omitted), one participant provided non-codable responses (e.g., brief answers such as “nothing”). Conversely, several participants provided multiple codable responses, resulting in a total of 290 codable responses included in the thematic analysis. | Results – Demographics |
| Setting | 14. Setting of data collection | Online (remote survey) | Methods – Settings |
|  | 15. Presence of non-participants | Not applicable (self-administered survey) | N/A |
|  | 16. Description of sample | Gender, age, years of experience, certification, client groups reported | Results – Demographics, Table 2 |
| Data collection | 17. Interview guide | Open-ended survey questions (e.g., “applicability and areas for improvement”) | Methods – Measures |
|  | 18. Repeat interviews | Not applicable (single survey) | Methods – Design |
|  | 19. Audio/visual recording | Not applicable (text responses only) | N/A |
|  | 20. Field notes | None | N/A |
|  | 21. Duration | Approx. 20 minutes per survey | Methods – Settings |
|  | 22. Data saturation | No mention of saturation; instead, ≥4% frequency threshold was applied | Methods – Thematic analysis |
|  | 23. Transcripts returned | Not applicable (participants directly wrote responses) | N/R |
| Domain 3. Analysis and findings | | | |
| Data analysis | 24. Number of data coders | Three coders, full consensus coding | Methods – Thematic analysis |
|  | 25. Coding framework | Meaning unit segmentation → preliminary codes → iterative consensus coding and codebook updates → theme confirmation; ≥4% threshold. Intercoder reliability not calculated (consensus approach) | Methods – Thematic analysis |
|  | 26. Derivation of themes | Codes directly reflecting quantitative results (e.g., performance expectancy predicting adoption intention) were minimally incorporated into higher–level themes to avoid redundancy and focus on novel insights complementing the quantitative analysis. | Discussion – Strengths |
|  | 27. Software | QDA Miner | Methods – Analysis |
|  | 28. Participant checking | No member checking conducted | N/R |
| Reporting | 29. Quotations presented | Representative quotations with IDs (e.g., “P29”, “P176”) | Results – Thematic results |
|  | 30. Data and findings consistent | Quotes, codes, themes, and frequency tables aligned consistently | Results – Table 8 |
|  | 31. Clarity of major themes | Three major themes with nine subthemes clearly presented | Results – Thematic results |
|  | 32. Clarity of minor themes and diversity | Diverse perspectives cited; overlapping UTAUT elements reported only as frequencies | Results – Thematic results |
